# Spurious interaction as a result of categorization

**DOI:** 10.1186/s12874-019-0667-2

**Published:** 2019-02-07

**Authors:** Magne Thoresen

**Affiliations:** Centre for Biostatistics and Epidemiology, Department of Biostatistics, University of Oslo, P.O. Box 1122, Blindern, N-0317 Oslo, Norway

**Keywords:** Categorization, Dichotomization, Interaction, Regression, Measurement error

## Abstract

**Background:**

It is common in applied epidemiological and clinical research to convert continuous variables into categorical variables by grouping values into categories. Such categorized variables are then often used as exposure variables in some regression model. There are numerous statistical arguments why this practice should be avoided, and in this paper we present yet another such argument.

**Methods:**

We show that categorization may lead to spurious interaction in multiple regression models. We give precise analytical expressions for when this may happen in the linear regression model with normally distributed exposure variables, and we show by simulations that the analytical results are valid also for other distributions. Further, we give an interpretation of the results in terms of a measurement error problem.

**Results:**

We show that, in the case of a linear model with two normally distributed exposure variables, both categorized at the same cut point, a spurious interaction will be induced unless the two variables are categorized at the median or they are uncorrelated. In simulations with exposure variables following other distributions, we confirm this general effect of categorization, but we also show that the effect of the choice of cut point varies over different distributions.

**Conclusion:**

Categorization of continuous exposure variables leads to a number of problems, among them spurious interaction effects. Hence, this practice should be avoided and other methods should be considered.

## Background

It is common in epidemiological and medical research to categorize exposure variables measured on a continuous scale and treat them as categorical in the statistical analysis. The continuous variables can be dichotomized or they can be divided into more than two groups, the latter alternative allowing investigation of a possible dose-response relationship. Examples of this practice include Body Mass Index (BMI) categorized according to pre-defined values and nutritional intake categorized according to observed quintiles. There may be several reasons for this practice. The most common ones being that it makes the analysis and the interpretation easier; one avoids having to model the actual relationship between the exposure variables and the response, and that it mimics clinical practice where one typically divides patients into groups (hypertensive vs. normotensive, obese vs. non-obese).

A number of papers have appeared, both in the biostatistical [1–7], epidemiological [8–13] and psychological [14–16] literature, pointing to problems with this approach and arguing against it. Among the problems are loss of information and power, but also an increased risk of type I error if continuous confounder variables are categorized. Recently, also predictive performance of models with categorized predictors is criticized [7]. We will not repeat their arguments here, but rather point to a problem that has received much less attention; that categorization of continuous exposure variables may lead to spurious interaction effects in a multiple regression model against an outcome.

This problem was observed already in 1974, by Humphreys and Fleishman [17], in a simulation study of the behavior of the ANOVA model in situations with two categorized explanatory variables. Later, the same type of problem was also noted by Paunonen and Jackson [18] in an investigation of possible effect modification of the association between personality trait measures and resulting behavior. They noted that effect modification, or interaction, was often observed if the effect modifier in question was dichotomized and a stratified analysis was performed, while if the same association was investigated in a linear regression model, keeping the effect modifier on its original scale and introducing a product term, effect modification was less often observed. This observation led Bissonnette et al. [19] to perform a simulation study investigating the same problem. They also found that when they simulated a model with no interaction, dichotomized the potential effect modifier and then performed a stratified analysis, effect modification was often observed. However, no real understanding of or explanation for the findings was provided.

Maxwell and Delaney [14] carried out a formal analytical investigation of the effects of dichotomizing the explanatory variables in a linear regression model. Specifically, they showed that in a model with two correlated explanatory variables *X*_1_ and *X*_2_, if the true relationship between one of the explanatory variables and the outcome *Y* was non-linear, a spurious interaction effect appeared when dichotomizing *X*_1_ and *X*_2_ at the median. This result has later been referred to by a number of other authors who have discussed the practice of categorization [2, 16], and the non-linear nature of the relationship between the explanatory variable and the outcome seems to have been taken as the explanation of the spurious interaction.

In this paper we will look into this problem in some more detail. We have two exposure variables *X*_1_ and *X*_2_ that are correlated, and where both of them are dichotomized. The situation where only one of them is dichotomized follows directly. Furthermore, they are both to be related to an outcome variable *Y* by some regression model. Throughout we will assume that there is no interaction between *X*_1_ and *X*_2_. In much earlier work, it has been assumed that the variables have been categorized at the median. However, in many medical and epidemiological applications, it is more relevant to consider categorization at more extreme values, and / or at several cut points. This leads to some interesting findings.

In our analytical treatment of the problem, we take the regression model to be linear, and we assume normally distributed variables *X*_1_ and *X*_2_. However, the results apply to regression models and distributions in general. We have also carried out a small simulation study in order to explore the effects for different distributions. We will first give two examples to show the relevance of the problem.

### Illustration 1, height and lung function

The first illustration uses data on lung function collected among Norwegian medical students. Peak expiratory flow (PEF), l/min, was measured six times for each student; three times in sitting position and three times in standing position. We will be using the mean of the six measurements in this illustration. In addition to PEF, we also measured the height of the students (cm) and we have gender information. In total we have data from 377 students, and we will model PEF as a function of gender and height (centered). Running the simple linear regression model PEF = *β*_0_ + *β*_1_ × gender + *β*_2_ × height + *β*3 × gender × height + *ε* leads to the following estimated coefficients (SE): ${\widehat{\beta}}_0$= 556.7 l/min (9.2 l/min), ${\widehat{\beta}}_1$= − 129.7 l/min (11.0 l/min), ${\widehat{\beta}}_2$ = 3.7 (l/min)/cm (0.9 (l/min)/cm), ${\widehat{\beta}}_3$ = − 0.8 l/min (1.1 l/min). Using the conventional 5% significance level, we have clearly significant effects of gender and height, but no interaction. Next, we categorize height according to the gender specific 90th percentiles; 189 cm for men and 175 cm for women, coding zero if the subject is below the cut-off and one if above. We will then run the same linear model as above, but with the categorized version of height. This leads to the following estimated coefficients (SE): ${\widehat{\beta}}_0$=578.4 l/min (6.4 l/min), ${\widehat{\beta}}_1$ = − 168.8 l/min (8.1 l/min), ${\widehat{\beta}}_2$ = 64.7 l/min (17.3 l/min), ${\widehat{\beta}}_3$ = − 48.5 l/min (22.6 l/min). We notice that we have a significant interaction at the conventional 5% level (*p* = 0.03). This would indicate that the effect of being among the higher 10% is significantly lower among women than among men, and while being among the 10% highest males leads to an increased PEF of 64.7 l/min, the same increase is only 16.2 l/min for females.

### Illustration 2, myocardial infarction

The second example is taken from a huge Norwegian health survey. During the period from 1985 until 1999 the Norwegian government conducted health surveys inviting men and women in the age of 40–42 years to participate. We will be analyzing a subset of these data, collected during the period 1985 to 1994. We have measured, among other things, Body Mass Index (BMI) and systolic blood pressure (BP) on a total of 133,139 subjects. These subjects have been followed for on average 19 years, and death of myocardial infarction is registered through a linkage to the Norwegian Cause of Death Registry. We will restrict our analysis to subjects with BMI > 20 to avoid having to deal with obvious non-linearities. This leaves us with 132,150 subjects. Among these there were 2542 (1.9%) deaths. We fit a logistic regression model for the odds of death by myocardial infarction as a linear function of BMI (kg/m^2^) and BP (per 10 mmHg) and the interaction between BMI and BP. The estimated coefficients (SE) are 0.20 (0.04) for BMI, 0.61 (0.08) for BP, − 0.01 (0.003) for the interaction term and a constant term of − 14.06 (1.20). Due to the large sample size, all the estimated effects are clearly significant at the conventional 5% level. However, the estimated interaction term has no practical significance. Next, we divide the sample into two BMI groups (obese vs. non-obese) by making a cut-off at 30 kg/m^2^, and we divide the sample into two BP groups (hypertensive vs. normotensive) by making a cut-off at 140 mmHg. Based on the two categorized variables we run the same logistic model as above (main effects of BMI and BP and the interaction BMI × BP). The estimated coefficients are 0.81 (0.09) for BMI, 1.03 (0.04) for BP, − 0.41 (0.11) for the interaction and a constant term of − 4.43 (0.03). This would indicate that the effect of being obese varies between normotensive and hypertensive so that among normotensive subjects there is an odds ratio of death equal Exp(0.81) = 2.25, while among the hypertensive the effect of being obese is reduced to an odds ratio of Exp(0.81–0.41) = 1.49, a substantial difference.

Both examples show how categorization may lead to substantial changes in the interpretation of the data in practical data analysis.

## Methods

### Analytical developments, categorization in the bivariate normal situation

Assume we have a linear relationship between two exposure variables *X*_1_, *X*_2_, and an outcome variable *Y*, satisfying the linear regression equation.

1 $$ Y={\beta}_0+{\beta}_1{X}_1+{\beta}_2{X}_2+\varepsilon $$

where we assume $$ \left({X}_1,{X}_2\right)\sim \mathrm{N}\left(0,\Sigma \right),\kern0.5em \Sigma =\left[\begin{array}{cc}1& \rho \\ {}\rho & 1\end{array}\right]. $$ Notice that there is no interaction in the true model. Furthermore, assume ${\overset{\sim }{X}}_1=I\left({X}_1>{c}_1\right),\kern0.5em {\overset{\sim }{X}}_2=I\left({X}_2>{c}_2\right)$ where for simplicity we let *c*_1_ = *c*_2_ = *c*. Let us define ${\mu}_{ij}=E\left(Y|{\overset{\sim }{X}}_1=i,{\overset{\sim }{X}}_2=j\right),\kern0.5em i,j=0,1$. We have no interaction between ${\overset{\sim }{X}}_1$ and ${\overset{\sim }{X}}_2$ if and only if *μ*_00_ − *μ*_10_ = *μ*_01_ − *μ*_11_⇒ *μ*_11_ − *μ*_01_ − *μ*_10_ + *μ*_00_ = 0.

Based on model (1) we have

2 $$ {\mu}_{00}={\beta}_0+{\beta}_1E\left({X}_1|{X}_1\le c,{X}_2\le c\operatorname{}\right)+{\beta}_2E\left({X}_2|{X}_1\le c,{X}_2\le c\operatorname{}\right) $$

3 $$ {\mu}_{01}={\beta}_0+{\beta}_1E\left({X}_1\left|{X}_1\le c,{X}_2>c\right.\right)+{\beta}_2E\left({X}_2\left|{X}_1\le c,{X}_2>c\right.\right) $$

4 $$ {\mu}_{10}={\beta}_0+{\beta}_1E\left({X}_1\left|{X}_1>c,{X}_2\le c\right.\right)+{\beta}_2E\left({X}_2\left|{X}_1>c,{X}_2\le c\right.\right) $$

5 $$ {\mu}_{11}={\beta}_0+{\beta}_1E\left({X}_1\left|{X}_1>c,{X}_2>c\right.\right)+{\beta}_2E\left({X}_2\left|{X}_1>c,{X}_2>c\right.\right). $$

In order to further investigate the relationships of interest, we need to be able to calculate the conditional expectations that enter these expressions. Define *F*_00_ = *P*(*X*_1_ ≤ *c* ∩ *X*_2_ ≤ *c*), *F*_01_ = *P*(*X*_1_ ≤ *c* ∩ *X*_2_ *>c*), *F*_10_ = *P*(*X*_1_ > *c* ∩ *X*_2_ ≤ *c*),  *F*_11_ = *P*(*X*_1_ > *c* ∩ *X*_2_ > *c*),

the probabilities of belonging to each of the four combinations of ${\overset{\sim }{X}}_1,\kern0.5em {\overset{\sim }{X}}_2$. Due to symmetry,

$$ {\displaystyle \begin{array}{c}E\left({X}_1|{X}_1\operatorname{}>c,{X}_2>c\right)=E\left({X}_2|{X}_1\operatorname{}>c,{X}_2>c\right),\\ {}E\left({X}_1|{X}_1\operatorname{}\le c,{X}_2>c\right)=E\left({X}_2|{X}_1\operatorname{}>c,{X}_2\le c\right),\\ {}E\left({X}_1|{X}_1>c,{X}_2\le c\operatorname{}\right)=E\left({X}_2|{X}_1\le c,{X}_2>c\operatorname{}\right),\\ {}E\left({X}_1|{X}_1\le c,{X}_2\le c\operatorname{}\right)=E\left({X}_2|{X}_1\le c,{X}_2\le c\operatorname{}\right),\end{array}} $$

so in the following we will focus on the conditional expectations of *X*_1_. Regier and Hamdan [20], using the Mehler identity [21], gave the following identity:

6 $$ {F}_{11}E\left({X}_1\left|{X}_1>c,{X}_2>c\right.\right)=\varphi (c)\left[1-\Phi \left(\frac{c-\rho c}{\sqrt{1-{\rho}^2}}\right)\right]\left(1+\rho \right), $$

where *φ*(⋅) denotes the normal density function and Φ(⋅) denotes the corresponding cumulative distribution function.

As indicated by Regier and Hamdan [20], corresponding identities can be found by appropriate combinations of integrals and we have that

7 $$ {F}_{10}E\left({X}_1|{X}_1>c,{X}_2\le c\operatorname{}\right)=\varphi (c)-{F}_{11}E\left({X}_1|{X}_1>c,{X}_2>c\operatorname{}\right), $$

8 $$ {F}_{01}E\left({X}_1\left|{X}_1\le c,{X}_2>c\right.\right)=\rho \varphi (c)-{F}_{11}E\left({X}_1\left|{X}_1>c,{X}_2>c\right.\right), $$

9 $$ {F}_{00}E\left({X}_1\left|{X}_1\le c,{X}_2\le c\right.\right)=E\left({X}_1\right)-{F}_{11}E\left({X}_1\left|{X}_1>c,{X}_2>c\right.\right)-{F}_{01}E\left({X}_1\left|{X}_1\le c,{X}_2>c\right.\right)-{F}_{10}E\left({X}_1\left|{X}_1>c,{X}_2\le c\right.\right). $$

As mentioned, we have no interaction if *μ*_11_ − *μ*_01_ − *μ*_10_ + *μ*_00_ = 0. Using Eqs. (2), (3), (4), (5), this leads to

10 $$ \left({\beta}_1+{\beta}_2\right)\left(E\left({X}_1\left|{X}_1>c,{X}_2>c\right.\right.\right)+E\left({X}_1\left|{X}_1\le c,{X}_2\le c\right.\right)-E\left({X}_1\left|{X}_1\le c,{X}_2>c\right.\right)-\left.E\left({X}_1\left|{X}_1>c,{X}_2\le c\right.\right)\right)=0 $$

From this, it is immediately clear that we can still have a spurious interaction even in situations where one of the two exposure variables is not associated with the outcome (*β*_1_ or *β*_2_ equal zero) as the product in (10) can still be different from zero. Furthermore, no spurious interaction can take place if *β*_1_ =  − *β*_2_. However, this last point is a function of our highly symmetrical situation and hence less relevant.

Using Eqs. (6), (7), (8), (9) and the fact that in our example, *E*(*X*_1_) = 0 and *F*_01_ = *F*_10_, formula (10) can be written

11 $$ \left({\beta}_1+{\beta}_2\right)\left(1+\rho \right)\varphi (c)\left[\frac{1-\Phi \left(\frac{c-\rho c}{\sqrt{1-{\rho}^2}}\right)}{F_{11}}-\frac{\Phi \left(\frac{c-\rho c}{\sqrt{1-{\rho}^2}}\right)}{F_{00}}-\frac{2\Phi \left(\frac{c-\rho c}{\sqrt{1-{\rho}^2}}\right)-1}{F_{01}}\right]=0 $$

We have to remember that *F*_11_, *F*_00_ and *F*_01_ are also functions of *c* and *ρ*. Solving this (numerically) leads to *ρ* = 0 and / or *c* = 0, in addition to *β*_1_ =  − *β*_2_ (and the degenerate solution *ρ* =  − 1 ). That is, we have no interaction if *X*_1_ and *X*_2_ are uncorrelated (*ρ* = 0) or if we split at the median (*c* = 0). Otherwise, categorization leads to spurious interaction. By the same arguments one can show that a spurious interaction will also appear if only one of the two variables *X*_1_ and *X*_2_ are categorized, or if *X*_1_ and *X*_2_ are split into more than two categories.

One should remember that Eq. (11) is only relevant for our specific situation, with normally distributed variables and common cut point *c*. The important message here is that the equation is true in only very few situations, which means that with a few exceptions, categorization leads to spurious interaction. This general message is true also for other distributions.

**Fig. 1:**
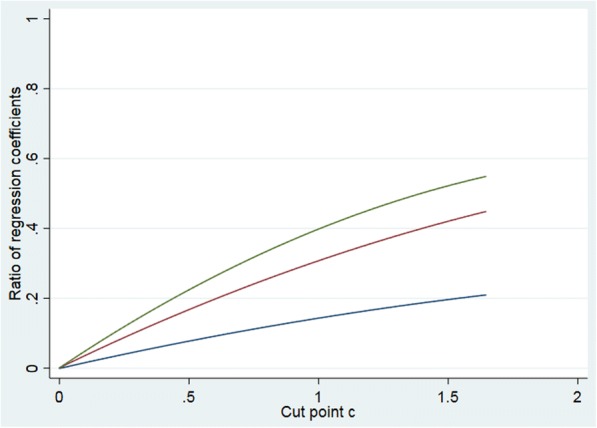
The figure gives the absolute value of the ratio between ${\overset{\sim }{\beta}}_3$ and ${\overset{\sim }{\beta}}_i,\kern0.5em i=1,2$ for *ρ* = 0.2 (blue line), 0.5 (red line) and 0.7 (green line) as a function of the cut point *c* when *β*_1_ = *β*_2_ = 1

**Fig. 2:**
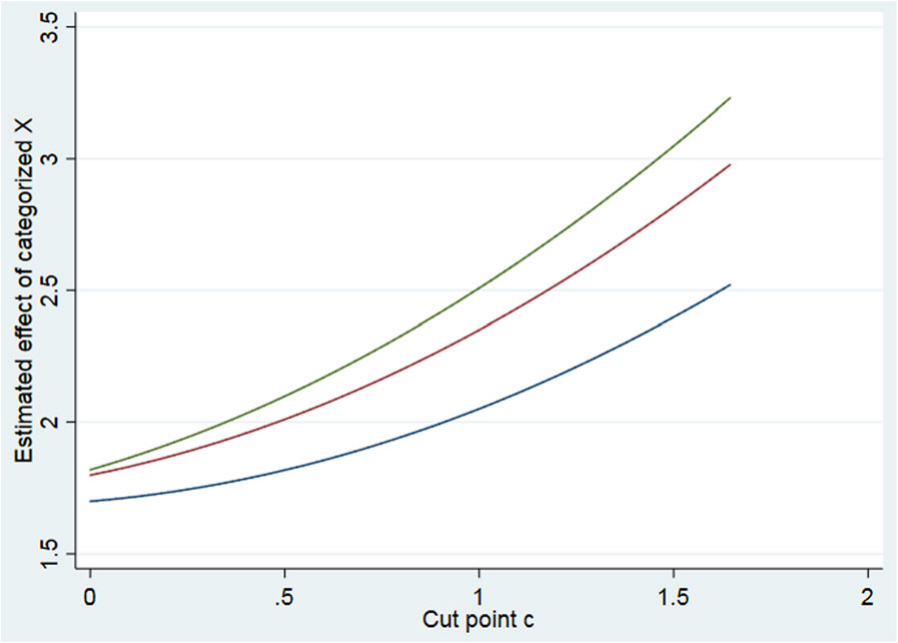
The figure gives ${\overset{\sim }{\beta}}_2$ for *ρ* = 0.2 (blue line), 0.5 (red line) and 0.7 (green line) as a function of the cut point *c.* True *β*_2_ = *β*_1_ = 1

Having established the presence of spurious interaction, it is of interest to investigate the potential size of the problem. Based on the model above, we can investigate the size of the induced interaction term relative to main effects of the categorized versions of *X*_1_, *X*_2_. Assume we fit a model $Y={\overset{\sim }{\beta}}_0+{\overset{\sim }{\beta}}_1{\overset{\sim }{X}}_1+{\overset{\sim }{\beta}}_2{\overset{\sim }{X}}_2+{\overset{\sim }{\beta}}_3{\overset{\sim }{X}}_1{\overset{\sim }{X}}_2+\overset{\sim }{\varepsilon }$ where ${\overset{\sim }{X}}_1,{\overset{\sim }{X}}_2$ are coded 0, 1. It is easy to show that ${\overset{\sim }{\beta}}_2$ is given by *μ*_01_ − *μ*_00_ where *μ*_00_ and *μ*_01_ are given in (2) and (3), and we have already established that ${\overset{\sim }{\beta}}_3$ is given by *μ*_11_ − *μ*_01_ − *μ*_10_ + *μ*_00_ . We can use this to investigate the size of ${\overset{\sim }{\beta}}_3$ relative to ${\overset{\sim }{\beta}}_2$ for different choices of *β*_1_, *β*_2_, the cut point *c* and the correlation *ρ*. Figure 1 gives the absolute value of ${\overset{\sim }{\beta}}_3/{\overset{\sim }{\beta}}_2$ for varying *c* and *ρ*, for *β*_1_ = *β*_2_ = 1. We observe that with increasing cut points, the influence of the induced product term becomes substantial, even for moderate values of the correlation *ρ*. In this case, the ratio of ${\overset{\sim }{\beta}}_3$ to ${\overset{\sim }{\beta}}_1$ will of course be the same as to ${\overset{\sim }{\beta}}_2$. As a bi-product we can also study the size of ${\overset{\sim }{\beta}}_1$ and ${\overset{\sim }{\beta}}_2$ as a function of the same cut point *c* and correlation *ρ* (Fig. 2), and we observe that the estimated effects increase quite rapidly with the more extreme cut points and correlations.

### Interpretation

Categorization of a continuous variable can be seen as an extreme form of measurement error. If there is measurement error in *X*_1_ and / or *X*_2_, and the error in *X*_1_ is differential with respect to *X*_2_ (meaning the measurement error in *X*_1_ varies with *X*_2_) or vice versa, it can be shown that this leads to an induced interaction between the observed versions of *X*_1_, *X*_2_ in a regression model [22–25]. In our case, the measurement error can be characterized by the reliability of the dichotomized variable ${\overset{\sim }{X}}_i$, relative to the continuous variable *X*_*i*_, *i* = 1, 2, as measured by the point-biserial correlation. If *X*_*i*_ is normally distributed, this correlation is given by $h/\sqrt{pq}$, where *h* denotes the ordinate of the normal curve at the cut point and *p* and *q* denote the proportion of the population (or probability mass) above and below the cut point. It is easily seen that this correlation will vary with the other variable *X*_*j*_, *i* ≠ *j*, as *p* and *q* will vary with *X*_*j*_. Hence, the measurement error is differential and an induced interaction is to be expected.

Another way of looking at the same problem is as a problem of residual confounding. If we have a situation with a confounding variable where the effect of the confounder is not properly adjusted for, we are left with some residual confounding. This is exactly what is happening when a confounder is categorized. It is well known that this leads to biased estimates of exposure - outcome associations. In particular, Marshall and Hastrup [26] showed through simulations that such residual confounding can lead to apparent effects of variables that are strongly correlated to the confounder, but which in reality bear no association with the outcome. Marshall and Hastrup termed this effect “*resonance of strong confounders*”. Important for our investigation, differences in residual confounding across strata may lead to spurious interaction [27].

### Simulations

To illustrate our findings and to further explore the effects of dichotomization in different situations we conducted a small simulation study. Notice that our main focus here has been to investigate the qualitative aspects of the induced interactions, and hence, we used a rather large sample size to minimize randomness.

We simulated *X*_1_, *X*_2_ according to three different distributions; standard bivariate normal, uniform [0,1], and chi-square with 2 df. We let the correlation between *X*_1_ and *X*_2_, *ρ*, vary over 0.2, 0.5, and 0.7, and we categorized at the 60th and the 80th percentiles, respectively. Furthermore, we simulated a response *Y* according to the following model, *Y* = *β*_0_ + *β*_1_*X*_1_ + *β*_2_*X*_2_ + *ε* with *ε* normally distributed with zero expectation and variance *σ*^2^ and independent of *X*_1_, *X*_2_. We then fit a model $Y={\overset{\sim }{\beta}}_0+{\overset{\sim }{\beta}}_1{\overset{\sim }{X}}_1+{\overset{\sim }{\beta}}_2{\overset{\sim }{X}}_2+{\overset{\sim }{\beta}}_3{\overset{\sim }{X}}_1{\overset{\sim }{X}}_2+\overset{\sim }{\varepsilon }$ where ${\overset{\sim }{\beta}}_3$ is an interaction parameter. In all our simulations we let *β*_0_ = 0 and *β*_1_ = *β*_2_ = 1. Furthermore, we let the residual variance *σ*^2^ vary over the distributions in such a way that Corr(*Y*, *X*_*i*_) = 0.3, *i* = 1, 2, when *ρ* = 0.5. The correlation *ρ* was then varied without changing *σ*^2^. The results of these simulations are given in Table 1. In addition, we repeated the situation with *ρ* = 0.7 and *X*_1_,  *X*_2_ categorized at the 80th percentile, but now with *β*_*i*_ = 2 for *i* = 1, 2. Further, we simulated the same situation once more with *β*_1_ = 1 and *β*_2_ = 0. The results of these simulations are given in Table 2.

**Table 1:** Results of the simulation study

		Normal	Uniform	Chi-square
60th percentile *ρ* = 0.2	${\widehat{\overset{\sim }{\beta}}}_1$	1.74 (0.11)	0.54 (0.04)	2.99 (0.25)
	${\widehat{\overset{\sim }{\beta}}}_2$	1.73 (0.11)	0.54 (0.04)	3.00 (0.26)
	${\widehat{\overset{\sim }{\beta}}}_3$	−0.06 (0.20)	− 0.03 (0.06)	0.63 (0.40)
60th percentile *ρ* = 0.5	${\widehat{\overset{\sim }{\beta}}}_1$	1.89 (0.14)	0.59 (0.04)	2.81 (0.28)
	${\widehat{\overset{\sim }{\beta}}}_2$	1.88 (0.14)	0.59 (0.04)	2.83 (0.29)
	${\widehat{\overset{\sim }{\beta}}}_3$	−0.16 (0.21)	− 0.08 (0.06)	1.38 (0.43)
60th percentile *ρ* = 0.7	${\widehat{\overset{\sim }{\beta}}}_1$	1.95 (0.15)	0.62 (0.04)	2.60 (0.31)
	${\widehat{\overset{\sim }{\beta}}}_2$	1.94 (0.15)	0.61 (0.04)	2.63 (0.32)
	${\widehat{\overset{\sim }{\beta}}}_3$	−0.24 (0.22)	− 0.12 (0.07)	1.82 (0.48)
80th percentile *ρ* = 0.2	${\widehat{\overset{\sim }{\beta}}}_1$	1.96 (0.14)	0.57 (0.04)	4.22 (0.27)
	${\widehat{\overset{\sim }{\beta}}}_2$	1.96 (0.14)	0.57 (0.04)	4.23 (0.28)
	${\widehat{\overset{\sim }{\beta}}}_3$	−0.23 (0.26)	− 0.11 (0.07)	0.25 (0.55)
80th percentile *ρ* = 0.5	${\widehat{\overset{\sim }{\beta}}}_1$	2.22 (0.15)	0.67 (0.04)	4.35 (0.31)
	${\widehat{\overset{\sim }{\beta}}}_2$	2.22 (0.16)	0.67 (0.05)	4.35 (0.32)
	${\widehat{\overset{\sim }{\beta}}}_3$	−0.58 (0.25)	− 0.27 (0.07)	0.50 (0.54)
80th percentile *ρ* = 0.7	${\widehat{\overset{\sim }{\beta}}}_1$	2.35 (0.16)	0.74 (0.05)	4.35 (0.35)
	${\widehat{\overset{\sim }{\beta}}}_2$	2.36 (0.18)	0.74 (0.05)	4.34 (0.35)
	${\widehat{\overset{\sim }{\beta}}}_3$	−0.81 (0.26)	− 0.40 (0.08)	0.58 (0.56)

The true regression coefficients *β*_*i*_ = 1,  *i* = 1,  2. The table gives estimated regression coefficients with corresponding empirical standard errors in parentheses

**Table 2:** Results of the simulation study

		Normal	Uniform	Chi-square
80th percentile *ρ* = 0.7 *β*_1_ = 1, *β*_2_ = 0	${\widehat{\overset{\sim }{\beta}}}_1$	1.65 (0.16)	0.50 (0.05)	3.35 (0.35)
	${\widehat{\overset{\sim }{\beta}}}_2$	0.70 (0.18)	0.24 (0.05)	0.99 (0.35)
	${\widehat{\overset{\sim }{\beta}}}_3$	−0.40 (0.26)	− 0.20 (0.08)	0.29 (0.55)
80th percentile *ρ* = 0.7 *β*_i_ = 2, *i* = 1, 2	${\widehat{\overset{\sim }{\beta}}}_1$	4.72 (0.17)	1.48 (0.05)	8.69 (0.36)
	${\widehat{\overset{\sim }{\beta}}}_2$	4.72 (0.18)	1.48 (0.05)	8.68 (0.37)
	${\widehat{\overset{\sim }{\beta}}}_3$	−1.63 (0.27)	− 0.79 (0.08)	1.16 (0.61)

The table gives estimated regression coefficients with corresponding empirical standard errors in parentheses

In order to generate correlated variates from the three distributions, we generated bivariate normal variates with a specified correlation structure in the standard way. Furthermore, we generated uniform marginals by applying the standard normal cumulative distribution function to each of the normal variates. Finally, on the basis of a uniform variate *V*_*j*_, we can generate *X*_*j*_ ∼ chi-square with 2 df. by *X*_*j*_ = − 2 ln(*V*_*j*_) [28]*.* It is an easy task to adjust the pre-specified correlation structure so that the observed correlations are as one wish. This is done empirically, by running preliminary simulations. For each setting we ran 1000 simulations with a sample size of 10,000.

## Results

Tables 1 and 2 give the results of these simulations. There are some common trends, but also some interesting differences between the distributions. First, the interaction effect becomes stronger with increasing correlation for all the distributions. We also observe that, naturally, the interaction effect becomes stronger with increasing main effects (the difference between Table 1 and the second situation in Table 2). In general, the normal distribution and the uniform distribution behave very similar. We see a stronger interaction effect with the more extreme cut-off (80th percentile vs. 60th percentile). In the skewed chi-square distribution, we observe the opposite; the stronger interaction effects appear with a cut-off at the 60th percentile. Furthermore, for the normal- and uniform distributions, the estimated interaction parameter has a negative sign, while for the chi-square distribution it has a positive sign. Finally, we confirm the result from the theoretical calculations, that even in the situation with *β*_2_ = 0, a spurious interaction appear.

If we look to the estimated standard errors, the interaction parameter becomes significant with a cut-off at the 60th percentile for the chi-square distribution in both Table 1 and Table 2, with an exception of the situation with the weaker correlation *ρ* = 0.2 in Table 1. For the other two distributions, we need in general to go to the more extreme cut-off (80th percentile) to find significant effects. Here, however, there are few significant effects for the chi-square distribution. It should be mentioned that categorization in general will lead to efficiency loss and the power to detect interaction effects will be low. For a general treatment of this topic, see [29].

To explain our findings, it will again be instructional to look at the problem as a measurement error problem. For the normal distribution, it is easy to show that the correlation between the continuous variable and the categorized version of the same variable is at its maximum with a cut-off at the median value, and it is then decreased with more extreme cut-off values. This means that there is more measurement error with the more extreme cut-off values. Based on the simulations, one can show that this is also true for the uniform distribution. However, also based on the simulations, for the chi-square distribution there is more measurement error with a cut-off at the 60th percentile than with a cut-off at the 80th percentile, as measured by the point-biserial correlation. This explains why we find the stronger interaction effects with a cut-off at the 60th percentile for the chi-square distribution, and with a cut-off at the 80th percentile for the two other distributions, as there will be more residual confounding, and hence more room for “resonance” in situations with more measurement error.

Furthermore, let us look into the differentiality of the measurement error. Thinking to the standard measurement error situation, with *β*_1_ and *β*_2_  >  0 we will have a positive interaction when there is less attenuation in the effect of *X*_1_ for higher values of *X*_2_ (and opposite), meaning the measurement error decreases with increasing *X*_2_. Again based on the simulated data, we can look at the measurement error (the point-biserial correlation) of *X*_1_ for ${\overset{\sim }{X}}_2=0$ vs. ${\overset{\sim }{X}}_2=1$. For the normal- and the Uniform distribution, we find more measurement error in *X*_1_ for the lower category of ${\overset{\sim }{X}}_2$, while for the chi-square distribution, there is more measurement error in *X*_1_ for the higher category of ${\overset{\sim }{X}}_2$. This explains why the sign of the interaction term differs between the distributions.

### Collapsing categorical covariates

We will briefly mention another consequence of this type of induced interaction. It is well known that tests of interaction usually have rather low power. When modelling an interaction between categorical exposure variables that takes more than two categories, a number of extra parameters have to be introduced. A common advice is then to combine exposure groups in order to decrease the number of extra parameters and hence, increase power (see e.g. Kirkwood & Sterne, Ch. 29.5) [30]. However, by doing this we will again run the risk of introducing a spurious interaction, which can be easily realized by the following illustration.

Assume we have one binary exposure variable *X*_1_ (e.g. gender) and one categorical exposure variable *X*_2_ with four categories. These two exposure variables are to be related to a binary outcome (diseased / not diseased). Assume the data look as in Table 3.

**Table 3:** Illustration of the effect of collapsing exposure categories

*X*_1_=1					*X*_1_=2				
*X*_2_	Diseased	Not diseased	Total	RR	*X*_2_	Diseased	Not diseased	Total	RR
1	20	380	400	1.0	1	5	95	100	1.0
2	30	270	300	2.0	2	20	180	200	2.0
3	30	170	200	3.0	3	45	255	300	3.0
4	20	80	100	4.0	4	80	320	400	4.0

The table gives the true situation

As seen from the table, there is no interaction between *X*_1_ and *X*_2_, as the association between *X*_2_ and the outcome as measured by the relative risk (RR) is constant across the two levels of *X*_1_. Next, we will combine categories 1 and 2, and 3 and 4 of *X*_2_, producing a new variable ${\overset{\sim }{X}}_2$ taking only two categories. This leads to Table 4.

**Table 4:** Illustration of the effect of collapsing exposure categories

*X*_1_=1					*X*_1_=2				
${\overset{\sim }{X}}_2$	Diseased	Not diseased	Total	RR	${\overset{\sim }{X}}_2$	Diseased	Not diseased	Total	RR
1	50	650	700	1.00	1	25	275	300	1.00
2	50	250	300	2.33	2	125	575	700	2.14

The table gives the observed situation after collapsing

As observed, the association between ${\overset{\sim }{X}}_2$ and the outcome now differs between the two levels of *X*_1_ and a spurious interaction is introduced. Although the effect is not particularly strong in this example, an apparent increase in power may partially be due to such an induced interaction.

## Discussion

We have given yet another argument to why continuous explanatory variables should not be categorized when entered into a regression model. If we have correlated exposure variables and categorize, this may lead to spurious interactions in the regression model. Furthermore, we have given an interpretation of this as a measurement error problem.

Statistical interaction is scale dependent, both with regard to model and measurement. An additive effect on a linear scale may appear as multiplicative on a transformed scale, like the logit. In the same way, any monotone transformation of the measurement scales of *X*_1_, *X*_2_ may lead to interactions. Hence, statistical interactions need to be interpreted relative to the scales on which they appear. As such, the main problem with the type of interaction discussed in the present work is not its existence but the fact that it is typically interpreted relative to its original measurement scale.

Our formal development has been within the framework of the linear regression model. However, based on the considerations above, it is easy to realize that this holds also for other regression models. Indeed, we have shown the appearance of such an interaction effect in logistic models through an example.

The practical implication of this is that whenever an interaction effect appears in an analysis based on categorized explanatory variables, the categorization itself should be considered as a possible explanation. However, one should also be aware that such an induced interaction may counteract any possible true interaction present in the data, and hence, mask this true interaction. It should be mentioned that in a practical data analysis, one will need a rather large sample size or strong effects for these interactions to appear as statistically significant.

## Conclusion

In summary, categorization of continuous variables should be avoided. It leads to a number of problems, including biased estimates, loss of power, inflated type-I error, and spurious interaction effects. If the true effect of the exposure variable(s) in question cannot be easily modelled by classical parametric models, non-parametric regression methods should be preferred in order to avoid the above-mentioned problems and to gain insight into the underlying relationship. If one choose to categorize despite such warnings, it is generally preferable to categorize into more than two groups in order to minimize the information loss.

## Abbreviations

ANOVA: Analysis of variance
Corr: Correlation
Df: Degrees of freedom
Exp(.): The exponential function
l/min: Liters per minute
SE: Standard error
